# METTL3-Dependent N6-Methyladenosine Modification Programs Human Neural Progenitor Cell Proliferation

**DOI:** 10.3390/ijms242115535

**Published:** 2023-10-24

**Authors:** Yuan Zhao, Jianguo Li, Yilin Lian, Qian Zhou, Yukang Wu, Jiuhong Kang

**Affiliations:** 1 Clinical and Translational Research Center of Shanghai First Maternity and Infant Hospital, Shanghai Key Laboratory of Maternal Fetal Medicine, School of Life Sciences and Technology, Tongji University, Shanghai 200092, China; zhao1212yuan@163.com (Y.Z.); lijianguo@tongji.edu.cn (J.L.); 2011407@tongji.edu.cn (Y.L.); zhouqian0415@foxmail.com (Q.Z.); 2 Shanghai Key Laboratory of Signaling and Disease Research, Frontier Science Center of Stem Cell Research, National Stem Cell Translational Resource Center, School of Life Sciences and Technology, Tongji University, Shanghai 200092, China

**Keywords:** METTL3, hESCs, hNPCs, neural differentiation, proliferation, m^6^A, SLIT2

## Abstract

METTL3, a methyltransferase responsible for N6−methyladenosine (m^6^A) modification, plays key regulatory roles in mammal central neural system (CNS) development. However, the specific epigenetic mechanisms governing human CNS development remain poorly elucidated. Here, we generated small−molecule−assisted shut−off (SMASh)−tagged hESC lines to reduce METTL3 protein levels, and found that METTL3 is not required for human neural progenitor cell (hNPC) formation and neuron differentiation. However, METTL3 deficiency inhibited hNPC proliferation by reducing *SLIT2* expression. Mechanistic studies revealed that METTL3 degradation in hNPCs significantly decreased the enrichment of m^6^A in *SLIT2* mRNA, consequently reducing its expression. Our findings reveal a novel functional target (SLIT2) for METTL3 in hNPCs and contribute to a better understanding of m^6^A−dependent mechanisms in hNPC proliferation.

## 1. Introduction

METTL3 (methyltransferase−like 3, also termed MTA70), a methyltransferase responsible for N6−methyladenosine (m^6^A) modification, plays key regulatory roles in RNA stability, splicing and translation, and is involved in the cell differentiation and reprogramming, embryonic development and tumor progression [1,2,3,4,5,6]. Recent studies have indicated that METTL3 exerts significant and multifaceted biological functions within the neuronal system. For example, deletion of *METTL3* in mouse hippocampus has been shown to diminish long−term memory integration [7]. Depletion of *METTL3* in adult neural stem cells (aNSCs) not only inhibits neuronal development and skews the differentiation of aNSCs more toward glial lineage, but also impacts the morphological maturation of newborn neurons in the adult brain [8]. However, few studies have thus far explored the role of METTL3 in human central neural system (CNS) development.

Mammal CNS development is a sequential and highly organized process that involves neural progenitor cells (NPCs) differentiating into various subtypes of neurons and glial cells [9]. NPCs share the characteristics of stem cells, possessing the capacity for both proliferation and differentiation into specialized progeny [10]. Proper proliferation and differentiation of NPCs are essential for preserving the neural progenitor pool and supporting brain development [11,12,13,14]. Dysregulation of NPC proliferation and differentiation can lead to severe neurodevelopment disorders, including microcephaly and autism spectrum disorder [15,16,17,18]. Yoon et al. have discovered that knockout *METTL14* in mouse brains extends the cell cycle of radial glia cells and prolongs cortical neurogenesis into postnatal stages [19]. Using the conditional knockout mouse model, Cao et al. have found that deletion of *FTO* in aNSCs enhances the proliferation and neuronal differentiation of aNSCs both in vitro and in vivo, but that over the long term the specific deletion of *FTO* hinders adult neurogenesis and neuronal development [20]. However, exploring the mechanisms underlying human CNS development remains challenging because of the absence of an appropriate model system.

Neural specification of human pluripotent stem cells (hPSCs) provides a valuable model to investigate human CNS development at the molecular and cellular levels [9,21,22,23]. Our previous research has demonstrated that EZH2, the methyltransferase for H3K27me3, specifies the normal neural fate decision by suppressing the competing meso/endoderm program [24]. Another study has shown that deletion of KDM6s does not inhibit NPC formation from hESCs. However, NPCs deficient in KDM6s display limited proliferation capacity and are unable to undergo differentiation into glial cells and neurons [25]. To date, attempts to create hESCs devoid of METTL3 have not yielded success, leaving the role of METTL3 in human CNS development unknown.

Here, we generated small−molecule−assisted shut−off (SMASh)−tagged hESC lines to diminish METTL3 protein, and found that METTL3 is not required for human neural progenitor cell (hNPC) formation and neuron differentiation, but the proliferation of hNPCs decreases with METTL3 deficiency by reducing slit guidance ligand 2 (*SLIT2*) expression. Mechanistic studies revealed that METTL3 degradation in hNPCs significantly decreased the enrichment of m^6^A in *SLIT2* mRNA, consequently reducing its expression. Our findings identified SLIT2 as a functional target of METTL3 in hNPCs.

## 2. Results

### 2.1. Generation of METTL3−SMASh−Tagged hESCs

**Figure 1:**
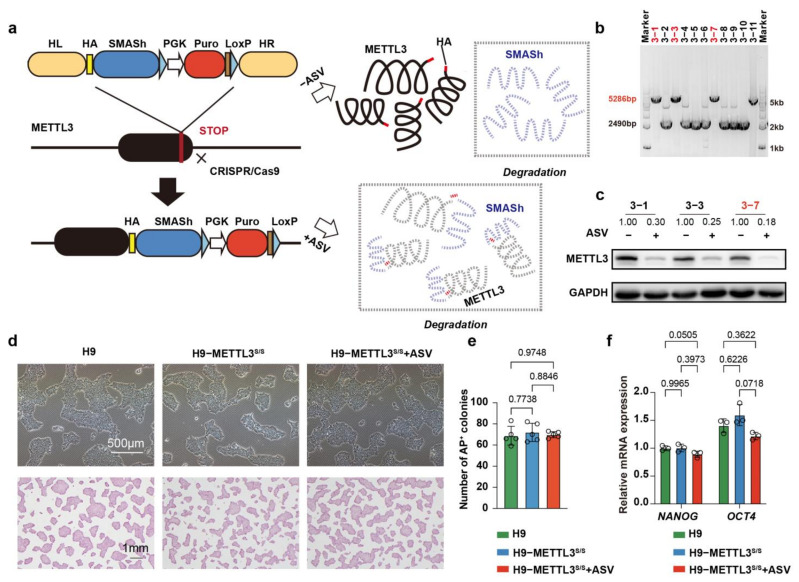
Generation of METTL3^s/s^ hESCs. (**a**) Schematic overview of generation of METTL3^s/s^ hESCs and ASV induced endogenous METTL3 protein degradation. hESCs, human embryonic stem cells. (**b**) PCR genotyping to identify H9−METTL3s/s hESC colonies. Homozygous colonies highlighted in red. The marker is a 1 kb ladder. (**c**) Western blot analysis of H9−METTL3s/s hESCs after ASV treatment for 4 days. GAPDH was probed as an internal loading control. (**d**) Morphology and alkaline phosphatase (AP) staining of H9 hESCs, H9−METTL3s/s hESCs and H9−METTL3s/s hESCs after ASV treatment for 10 days. Scale bar, 500 μm and 1 mm. (**e**) Quantitative data representing the number of AP−positive colonies of H9 hESCs, H9−METTL3s/s hESCs and H9−METTL3s/s hESCs after ASV treatment for 10 days. (**f**) qRT−PCR analysis showing the expression levels of the pluripotent genes (*NANOG* and *OCT4*) in H9 hESCs, H9−METTL3s/s hESCs and H9−METTL3s/s hESCs after ASV treatment for 10 days. Data represent mean ± SD (*n* = 3, two−way ANOVA).

To gain insights into the role of METTL3 in cell fate determination, we created SMASh tagged hESCs through CRISPR/Cas9 genome editing in conjunction with homologous recombination [26] (Figure 1a). To confirm the SMASh inserted into hESCs, we amplified the METTL3 genome using PCR, and the results showed that we obtained three METTL3−SMASh−tagged hESC (H9−METTL3^s/s^) lines (Figure 1b). To test whether SMASh could effectively reduce the expression level of METTL3 in hESCs, we treated the cells with 2 µM asunaprevir (ASV) for 4 days, and found that ASV significantly reduced the protein level of METTL3 (Figure 1c). Thus, we have established H9−METTL3^s/s^ hESCs.

To confirm the role of METTL3 in the maintenance of hESCs, we examined the morphology of cells and quantified the number of colonies using alkaline phosphatase (AP) staining. We noted that the cells maintained a typical undifferentiated state and the number of AP positive colonies was similar for H9 hESCs and H9−METTL3^s/s^ after ASV administration (Figure 1d, e). Furthermore, we assessed the expression levels of pluripotency-related genes, and found that the expression levels of *OCT4* and *NANOG* were similar for H9 hESCs and H9−METTL3^s/s^ after ASV administration (Figure 1f). Together, these findings suggest that METTL3 is not essential for the maintenance of an undifferentiated state of hESCs.

### 2.2. METTL3^s/s^ hESCs Undergo Normal Neural Differentiation and Neuron Differentiation

**Figure 2:**
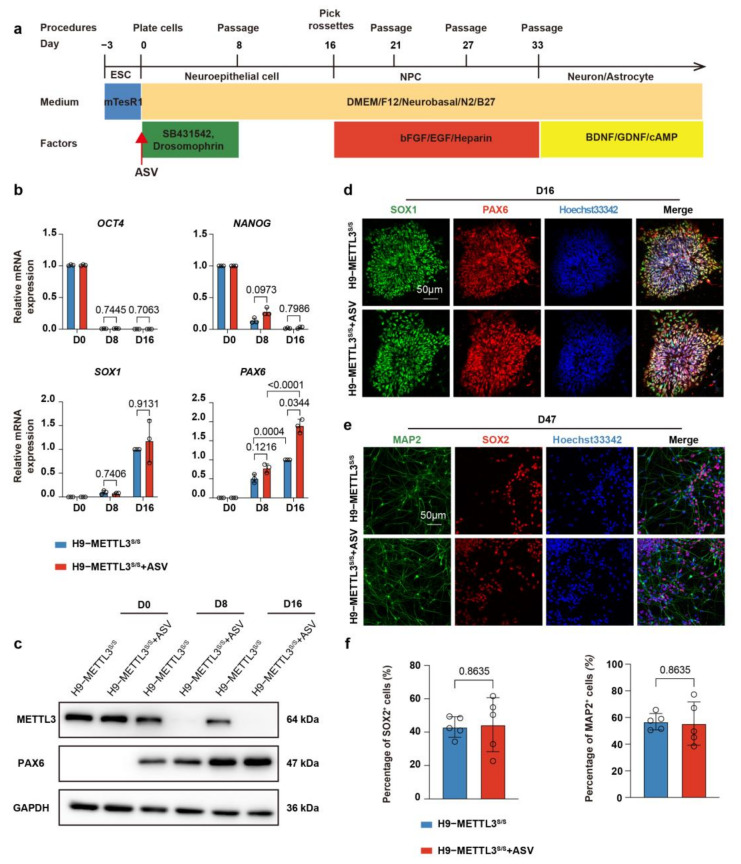
NPC specification and neuron differentiation of H9−METTL3^s/s^ hESCs. (**a**) Overview of the monolayer neural specification protocol for hESCs. hESCs were exposed to two SMAD inhibitors (5 μM SB431542 and 5 μM dorsomorphin) in the indicated defined medium for neural induction. The rosette−like hNPCs were harvested at day 16 (D16) and expanded as neurospheres. To initiate neuron differentiation, hNPCs were subsequently plated on Matrigel and maintained in the specified medium to induce spontaneous differentiation (refer to Section 4 for details). (**b**) qRT−PCR analysis showing the relative expression levels of the pluripotent genes (*NANOG* and *OCT4*) and the hNPC genes (*PAX6* and *SOX1*) in H9−METTL3s/s hESCs and H9−METTL3s/s hESCs after ASV treatment at D0, D8 and D16 of neural specification. Values represent mean ± SD (*n* = 3, two−way ANOVA). (**c**) Western blot analysis of H9-METTL3s/s hESCs and H9−METTL3s/s hESCs after ASV treatment for METTL3 and PAX6 at D0, D8 and D16 of neural specification. GAPDH was probed as an internal loading control. The quantitative results are shown in Supplementary Figure S1b. (**d**) Immunostaining images depicting the distributions of SOX1 (green) and PAX6 (red) in H9−METTL3s/s hESCs and H9−METTL3s/s hESCs after ASV treatment at D16 of neural specification. Scale bar, 50 μm. (**e**) Immunostaining images of H9−METTL3s/s hNPCs and H9−METTL3s/s hNPCs after ASV treatment for MAP2 (green) and SOX2 (red) at D47 of neural specification. Scale bar, 50 μm. (**f**) Quantitative data regarding the percentage of SOX2^+^ or MAP2^+^ cells in H9−METTL3s/s hNPCs and H9−METTL3s/s hNPCs after ASV treatment at day 47 of neural differentiation. Data represent mean ± SD (*n* = 5, unpaired *t* test).

To investigate the role of METTL3 in human CNS development, we performed neural specification of METTL3^s/s^ hESCs using a well−defined protocol involving dual inhibition of TGF_β_/BMP signaling and the EB method [21,27] (Figure 2a). Upon differentiation, with/without ASV treatment, we observed a significant suppression of the pluripotent genes *OCT4* and *NANOG*, alongside an upregulation of the NPC genes *PAX6* and *SOX1* at day 16 of differentiation (Figure 2b, Supplementary Figure S1a). As expected, ASV treatment significantly reduced the expression level of METTL3 at day 8 and 16 of differentiation process (Figure 2c, Supplementary Figure S1b,c). These data indicate that the decreased expression of METTL3 does not impede the transition from pluripotency to NPC differentiation in hESCs. In contrast to EZH2^−/−^ hESCs (used as a negative control) [24], METTL3^s/s^ hESCs, both treated and untreated with ASV, exhibited a rosette-like morphology, the typical NPC phenotype in hESC neural specification, while PAX6 positive cells and PAX6 protein levels remained consistently in both ASV−treated and untreated METTL3^s/s^ hESCs (Figure 2c,d, Supplementary Figure S1b,d–f). Additionally, immunostaining data demonstrated that the rosette-like cells derived from METTL3^s/s^ hESCs (with or without ASV) exhibited high expression of the canonical NPC marker SOX1 (Figure 2d, Supplementary Figure S1d,e). Together, these data demonstrate that METTL3 deficiency in hESCs does not hinder fate transition at the early stage of neural specification.

To further examine the role of METTL3 in hNPC differentiation, we initiated spontaneous neuron differentiation with the addition of BDNF, GDNF and cAMP (Figure 2a). After 14 days of culture, the hNPCs ceased proliferation and began differentiating into neurons, as indicated by immunostaining for neuron marker MAP2 and hNPC marker SOX2 (Figure 2e). We also found that ASV treatment did not influence the percentage of SOX2^+^ hNPCs and MAP2^+^ neurons or the percentage of NEUN^+^/TUJ1^+^ neurons (Figure 2e,f, Supplementary Figure S1g). In summary, our data confirm that METTL3 deficiency does not impede spontaneous neuron differentiation of hNPCs.

### 2.3. METTL3 Regulates hNPC Proliferation In Vitro

**Figure 3:**
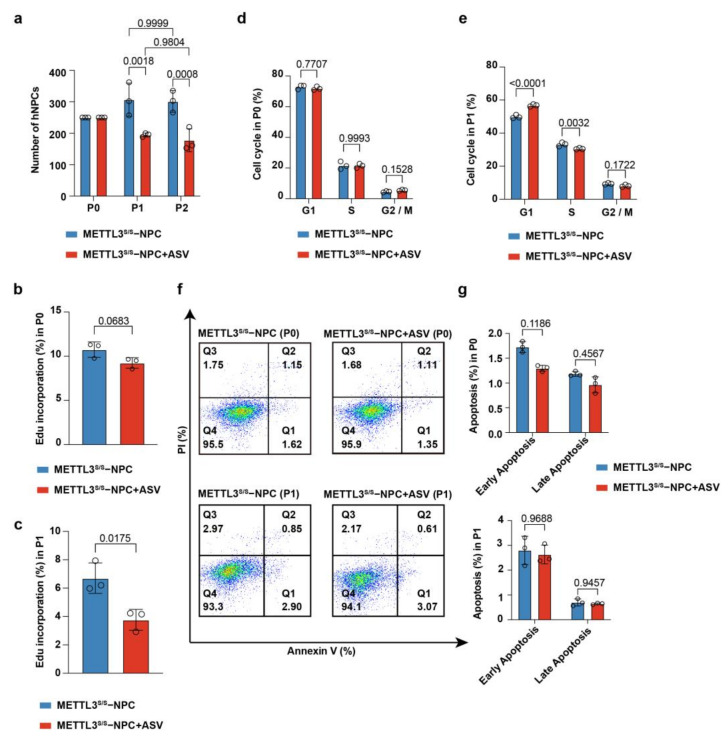
METTL3 is essential for proliferation of hNPCs. (**a**) Proliferation analysis of METTL3s/s hNPCs and METTL3s/s hNPCs after ASV treatment at different passages. Data represent mean ± SD (*n* = 3, two−way ANOVA). (**b**,**c**) EdU incorporation assay of METTL3s/s hNPCs and METTL3s/s hNPCs after ASV treatment at P0 (**b**) and P1 (**c**). Data represent mean ± SD (*n* = 3, unpaired *t* test). (**d**,**e**) Cell cycle assay of METTL3s/s hNPCs and METTL3s/s hNPCs after ASV treatment at passage 0 (P0) (**d**) and passage 1 (P1) (**e**). Values represent mean ± SD (*n* = 3, two−way ANOVA). (**f**,**g**) Apoptosis assay in METTL3s/s hNPCs and METTL3s/s hNPCs after ASV treatment at P0 and P1 (**f**). PI− and/or annexin V−positive cells were analyzed using FACS (**g**). Data represent mean ± SD (*n* = 3, two−way ANOVA).

NPCs have the potential to proliferate and differentiate [10,28]. To examine the role of METTL3 in hNPC proliferation, we isolated rosette−like hNPCs and cultured them as neurospheres in the specified medium with an equal initial cell count. During passaging, we found that upon administration of ASV the number of hNPCs reduced rapidly (Figure 3a). Consistently, the EdU incorporation of METTL3s/s hNPCs (treated with ASV) at P1 was lower than METTL3s/s hNPCs (Figure 3b,c). Additionally, as METTL3 levels decreased, there was a noticeable shift in the cell cycle, characterized by a decrease in the S phase and an increase in the G1 phase at P1 (Figure 3d,e). We then performed apoptosis analysis, with the results showing that METTL3 deficiency had no effect on the hNPC apoptosis (Figure 3f,g). Using the EB method, the number of neurospheres was reduced after ASV administration, while the neurosphere diameter was similar (Supplementary Figure S2a,b). The results revealed a reduction in the number of hNPCs following ASV administration. Taken together, our findings reveal that METTL3 is essential for hNPC proliferation.

### 2.4. METTL3 Deficiency Reduces m^6^A Modification on hNPCs-Related Genes

**Figure 4:**
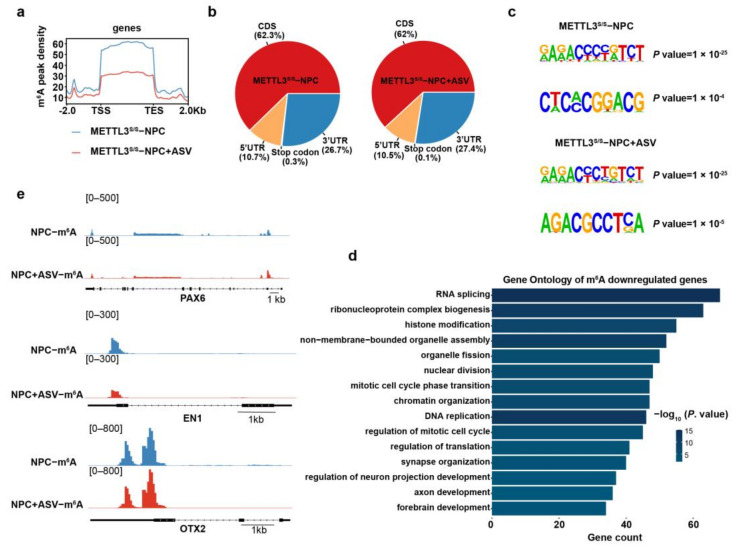
METTL3 deficiency reduces m^6^A modification on hNPCs genes. (**a**) Metagene profiles illustrating the distribution of m^6^A signals along a normalized transcript, divided into three non-overlapping segments: 5′ UTR, CDS, and 3′ UTR in METTL3s/s hNPCs and METTL3s/s hNPCs after ASV treatment. (**b**) Pie chart depicting the distribution of m^6^A sites in four regions of H9-METTL3s/s hNPCs and METTL3s/s hNPCs post ASV treatment. (**c**) Identification of consensus motifs on m^6^A peaks identified in METTL3s/s hNPCs and METTL3s/s hNPCs following ASV treatment. (**d**) Gene Ontology (GO) analysis focusing on m^6^A downregulated genes in METTL3s/s hNPCs after ASV treatment. (**e**) Genome browser snapshot displaying MeRIP-seq read density in exonic regions of representative hNPCs genes for METTL3s/s hNPCs and METTL3s/s hNPCs after ASV treatment.

METTL3 is the major methyltransferase that catalyzes the formation of m^6^A in mRNAs [29,30,31,32,33]. Thus, we performed methylated RNA immunoprecipitation sequencing (MeRIP−seq) in METTL3^s/s^ hNPCs with or without ASV. Bioinformatic analysis indicated an abundance of m^6^A peaks in 3′ untranslated regions (3′ UTR) (Figure 4a, b, Supplementary Figure S3a). Further analysis showed that, along with the METTL3 deficiency, m^6^A signals were downregulated near the transcription start site (TSS) and transcription end site (TES) (Figure 4a, Supplementary Figure S3a). Consistent with existing knowledge, these m^6^A peaks exhibited a distinct RRACH (R = G/A, H = A/C/U) consensus motif (Figure 4c). Additional Gene Ontology (GO) analysis revealed that the genes that lost m^6^A peaks were related to RNA splicing, histone modification, mitotic cell cycle phase transition and CNS development (for example, synapse organization, axon development and forebrain development) (Figure 4d, Supplementary Table S4). Consistently, the administration of ASV led to many known hNPCs genes such as PAX6, EN−1 and OTX2 reducing the m^6^A signals near 3′ UTR (Figure 4e). To draw a conclusion, METTL3 deficiency reduces m^6^A modification on certain hNPC genes.

### 2.5. SLIT2 Is the Functional Target of METTL3 in hNPCs

**Figure 5:**
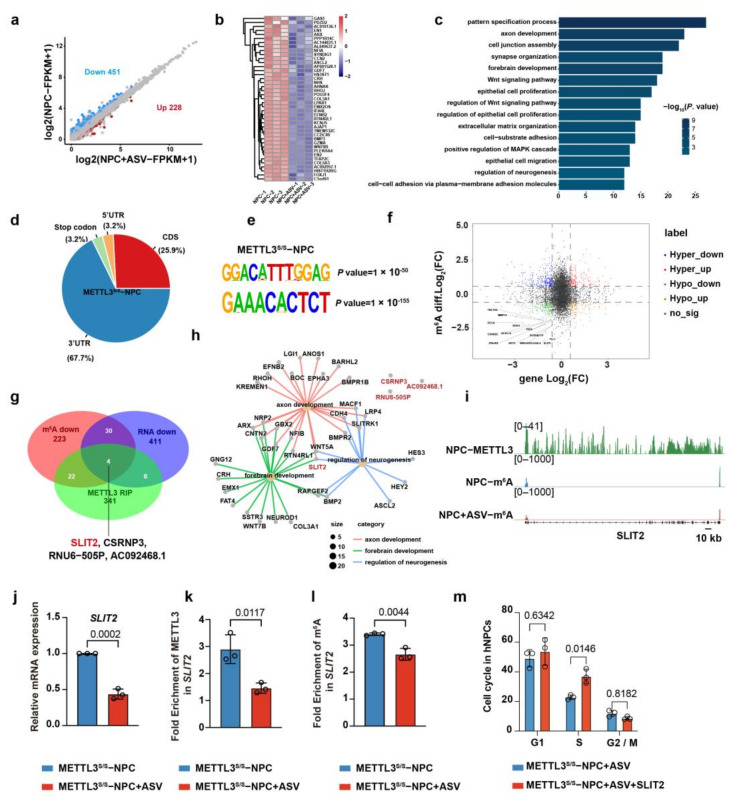
SLIT2 is the functional target of METTL3 in hNPC proliferation. (**a**) Volcano plot depicting differential expression genes (DEG) in METTL3s/s hNPCs and METTL3s/s hNPCs after ASV treatment. (**b**) Heatmap illustrating downregulated genes in METTL3s/s hNPCs after ASV treatment. (**c**) GO analysis focusing on downregulated genes in METTL3s/s hNPCs after ASV treatment. (**d**) Pie chart displaying the distribution of METTL3 signals across four regions of METTL3s/s hNPCs. (**e**) Identification of consensus motifs within METTL3 peaks identified in METTL3s/s hNPCs. (**f**) Volcano plot showing DEG and m^6^A−modified genes in METTL3s/s hNPCs and METTL3s/s hNPCs after ASV treatment. (**g**) Venn diagram representing METTL3-binding transcripts, m^6^A downregulated genes and downregulated genes in METTL3s/s hNPCs after ASV treatment. (**h**) Representation of GO terms related to biological process categories enriched in downregulated genes with RNA−seq. (**i**) Genome browser snapshot displaying RIP−seq and MeRIP−seq read density within exonic regions of SLIT2. (**j**) qRT−PCR analysis of the expression levels of *SLIT2* in METTL3s/s hNPCs and METTL3s/s hNPCs after ASV treatment. METTL3s/s hNPCs serve as control. Data represent mean ± SD (*n* = 3, unpaired *t* test). (**k**) RIP−qPCR analysis of the METTL3 enrichment level on *SLIT2* in METTL3s/s hNPCs and METTL3s/s hNPCs after ASV treatment. METTL3s/s hNPCs serve as control. Data represent mean ± SD (*n* = 3, unpaired *t* test). (**l**) MeRIP−qPCR analysis of the m^6^A enrichment level on *SLIT2* in METTL3s/s hNPCs and METTL3s/s hNPCs after ASV treatment. METTL3s/s hNPCs serve as control. Data represent mean ± SD (*n* = 3, unpaired *t* test). (**m**) Cell cycle assay comparing METTL3s/s hNPCs after ASV treatment and METTL3s/s hNPCs after ASV and SLIT2 recombination protein treatment at passage 1 (P1). Data represent the mean ± SD (*n* = 3, two−way ANOVA).

To further investigate the potential targets of METTL3 in hNPCs, we performed RNA sequencing (RNA−seq) in METTL3^S/S^ hNPCs treated with or without ASV. A total of 451 and 228 genes were found to be downregulated or upregulated, respectively, in hNPCs treated with ASV (Figure 5a). Intriguingly, GO analysis showed that genes downregulated in hNPCs treated with ASV were specifically enriched for pattern specification, central nervous system development (for example, axon development, synapse organization, forebrain development and neurogenesis) and epithelial cell proliferation, whereas the upregulated genes were closely related to non−neural development, such as ear development, kidney development and reproductive structure development (Figure 5b,c, Supplementary Figure S3b, Supplementary Tables S5 and S6). Furthermore, immunoprecipitation sequencing (RIP−seq) was performed to decipher the METTL3 landscape in normal hNPCs. Notably, METTL3 peaks were significantly abundant in 3′ UTR, consistent with m^6^A peaks (Figure 5d, Supplementary Figure S3c). Moreover, the signals of METTL3 peaks showed a clear RRACH consensus motif (Figure 5e).

To find the key target genes of METTL3 in hNPCs, we stratified the differential expression genes in METTL3^S/S^ hNPCs (treated with ASV) with m^6^A marking genes, and found that 56 downregulated genes were regulated by m^6^A methylation (Figure 5f,g); the top genes are listed in Supplementary Figure S4a. In combination with the METTL3−modified transcripts, we found that only four downregulated genes, SLIT2, CSRNP3, RNU6−505P and AC092468.1 were regulated by METTL3−modified m^6^A methylation (Figure 5g). Among these genes, RNU6−505P and AC092468.1 were pseudogenes, representing remnants of genes that have lost their biological functions. Our previous RNA−seq and qPCR showed that *SLIT2* manifested elevated expression levels in hNPCs, while *CSRNP3* was predominantly expressed during the hESCs−to−hNPCs transition (Supplementary Figure S4b,c). These results imply that *SLIT2* may be the target of METTL3 in hNPCs.

SLIT2 has crucial roles in organ development, including nervous system development), tumor progression, stem cell regulation, and cell proliferation [34,35,36,37,38,39]. GO term analysis showed that only SLIT2 was related to axon development, forebrain development and neurogenesis (Figure 5h). To further validate METTL3 regulation of *SLIT2* expression in an m^6^A−dependent manner, we performed qPCR, RIP−qPCR and MeRIP−qPCR in hNPCs (with or without ASV) (Figure 5i). The results showed that, along with METTL3 deficiency, the *SLIT2* expression level, fold enrichment of METTL3 and m^6^A in *SLIT2* were downregulated (Figure 5j–l). In addition, we found that recombinant human SLIT2 augmented the ratio of S phase in METTL3^S/S^ hNPCs treated with ASV (Figure 5m). Overall, these data indicate that SLIT2 is the functional target of METTL3 in hNPCs.

## 3. Discussion

METTL3 plays essential roles in the cell differentiation, reprogramming, embryonic development and tumor progression [2,4,40,41,42]. ESCs are an excellent model for investigating the early development of the nervous system and the regulatory mechanisms [24,25,43]. Knockout of *Mettl3* in mESCs fails to adequately terminate their naïve state, and blocks differentiation [44,45]. However, due to the failure of construction of the *METTL3* knockout hESCs, the effect of *METTL3* knockout on hESCs remains unclear. Here, we successfully construct METTL3-SMASh tagged hESCs. ASV treatment decreases the expression of METTL3 in METTL3-SMASh tagged hESCs, but does not affect the stem cell morphology and the expression levels of *OCT4* and *NANOG*. OCT4 and NANOG are recognized as pivotal components of the core pluripotency circuitry [46,47,48]. OCT4 (also known as OCT3) is the foremost pluripotency factor, and is the first transcription factor identified as a regulator of pluripotency [49,50,51,52,53]. Niwa et al. have found that the precise level of OCT4 determines three distinct fates of ESCs [52]. An increase in *OCT4* expression leads to differentiation into primitive endoderm and mesoderm [52]. Conversely, repression of *OCT4* results in loss of pluripotency and dedifferentiation to trophectoderm [52]. ESCs can maintain pluripotency only when the levels of *OCT4* expression remain normal. NANOG is a classic pluripotency-maintaining factor [54,55,56,57,58]. *NANOG* deficiency in ESCs leads to exit pluripotency and differentiated into extraembryonic endoderm [57]. Overexpressing *NANOG* in ESCs is sufficient for colonization and expansion of ESCs [58]. Our findings show that hESCs maintain their pluripotent state in the absence of METTL3 (Figure 1). These findings are consistent with previous observations of *METTL3* knockdown in hESCs [44]. These phenomena indicate that METTL3 plays distinct roles in hESCs and mESCs. Our cell lines provide a valuable platform for investigating the impact of varying METTL3 levels in human embryonic development, for example CNS development, cardiac development and hematopoiesis. Additionally, these cell lines serve as a foundational resource for exploring the functions of METTL3 at various stages of human embryonic development, leveraging the reversibility of drug−induced protein degradation.

METTL3 has important roles in the mammal CNS system [8,59,60]. Research has shown that cortical−specific conditional knockout *METTL3* in mice leads to an elevated population of intermediate progenitor cells (iPCs) and inhibits neuronal production [61]. The absence of *METTL3* alters neurite enrichment of a subset of mRNAs and reduces the localization of select methylated RNAs in mouse hippocampal neurons [22]. In *Xenopus laevis*, knockdown of *METTL3* results in anteriorization of neurulae and tailbud embryos, accompanied by the depletion of neural crest and neuronal cells [62]. Accumulated studies, however, have found that human neural development is not identical to the model animals; for example, PAX6 exhibits delayed expression in restricted mouse brain regions, in contrast to its uniform expression in early neuroepithelial cells in hESCs [43]. Our study indicates that METTL3 is essential for hNPC proliferation but not required for hNPC formation and differentiation (Figure 2 and Figure 3). These interesting findings suggest that the role of METTL3 in human neurodevelopment is species-specific and different from that of other model animals such as the mouse and *Xenopus laevis*.

METTL3 serves as the principal methyltransferase responsible for m^6^A modification, and thousands of studies have found that depletion of METTL3 apparently diminishes m^6^A signals [29,30,31]. Our MeRIP−seq analysis reveals that, concurrent with METTL3 degradation, numerous genes experience a loss of m^6^A peaks, while these m^6^A peaks exhibit a distinct RRACH consensus motif (Figure 4). Our results are consistent with established knowledge [42,63]. These observations imply that METTL3 plays a pivotal role as the primary methyltransferase in mediating m^6^A formation in hNPCs.

Our research reveals SLIT2 as a novel target of METTL3 in hNPCs (Figure 5). Previous studies have established the crucial involvement of SLIT2 in mouse axon guidance, hypothalamus development and neurogenesis [36,64,65,66]. Borrell et al. have demonstrated that the disruption of SLIT/ROBO signaling results in a reduction of ventricular zone (VZ) progenitors and a concurrent rise in iPCs [36]. Additionally, their work highlights the fact that ROBO receptors sustain the equilibrium of cortical progenitors by modulating the NOTCH pathway through controlling *Hes1* transcription [36]. Romanov et al. have revealed that loss of SLIT2/ROBO signaling adversely affects both the generation and positioning of periventricular dopamine neurons during hypothalamus development [65]. These studies investigate the downstream of SLIT2/ROBO signaling in CNS development. Our results imply that METTL3 functions as an upstream regulator of SLIT2/ROBO signaling in hNPCs.

In summary, our study has constructed the METTL3−SMASh−tagged hESCs, enabling the modulation of protein dosage from mild reduction to complete knock out in hESCs. Furthermore, our study demonstrates that METTL3 does not affect neural differentiation and neuron differentiation of hESCs, but is required for the proliferation of hESC−derived hNPCs, and SLIT2 is the functional target of METTL3 in hNPC proliferation. Thus, our study constructs the METTL3−deficient hESCs, offers a platform to study the role of METTL3 in human embryonic development, and elucidates the function and mechanism of METTL3 in human CNS development.

## 4. Materials and Methods

### 4.1. Cell Culture

We cultured the human embryonic stem cell lines H9 (Wi Cell) and the SMASh−tagged hESCs under feeder−free conditions on Matrigel (Corning, NY, USA) −coated plates in mTeSR1 medium (STEMCELL Technologies, VAN, Canada).

### 4.2. Construction of Genome Editing Vectors

The guide RNAs (gRNAs) for METTL3 were designed from https://cctop.cos.uni-heidelberg.de:8043 (accessed on 19 August 2020).

To create the SMASh donor vector, we PCR amplified the “PGK−Puro” sequence, the homologous recombination arms (HR arms) of METTL3, and the HA×3−SMASh DNA sequence. These fragments were then cloned into the pZERO vector (Tiangen, Beijing, China) using the Hieff Clone Plus Multi One−Step Cloning Kit (Yeasen, Shanghai, China).

### 4.3. Generation of SMASh Tagged hESCs

To construct SMASh tagged hESCs, electroporation was performed using the LONZA 4D−Nucleofector. The day before nucleofection, we added 10 μM ROCK inhibitor Y27632 (Selleck, Houston, TX, USA, S1049) to the medium. Subsequently, hESCs were digested using Accutase (Thermo Fisher, Waltham, MA, USA). A single nucleofection event utilized 10 μg pX330−sgRNA plasmid and 15 μg donor plasmid. All primer sequences are listed in Supplementary Information Table S2 (BioSune, Shanghai, China, Supplementary Information).

Puromycin (0.5 μg mL^−1^) was added between day 4 and day 8 after nucleofection, followed by an increase to 1 μg mL^−1^ for an additional 4 days. Two weeks after nucleofection, we selected 11 colonies for PCR and Western blot analysis.

### 4.4. Alkaline Phosphatase (AP) Staining

We conducted AP staining utilizing an Alkaline Phosphatase Staining Kit for Stem Cell (MKBio, Shanghai, China) in accordance with the manufacturer’s guidelines. The cells were rinsed twice with PBST, followed by fixation in fix solution at room temperature for 2–5 min. After washing three times with PBST, the cells were stained with staining buffer (Buffer A: Buffer B: Buffer C = 1:1:1) at room temperature for 5–15 min. Following three PBS washes, the cells were captured with a microscope (Olympus, Tokyo, Japan).

### 4.5. Asunaprevir (ASV) Administration and Neural Progenitor Cell (NPC) Formation from hESCs

ASV (Selleck, Houston, TX, USA, S4935) was initially dissolved in DMSO to create a 100 mM stock solution. Before use, this stock solution was further diluted to 10 mM with DMEM/F12 and subsequently incorporated into the culture medium to reach a final concentration of 2 μM. In control groups, an equivalent concentration of DMSO (0.002%) was added.

The monolayer neural specification of hESCs was conducted as described previously [25,67]. Briefly, hESCs were transitioned to N2B27 medium, consisting of a 1:1 mixture of DMEM/F12 and neurobasal (both from Gibco, Carlsbad, CA, USA), enriched with 0.5 × N2 (Gibco, Carlsbad, CA, USA), 0.5 × B27 (Gibco, Carlsbad, CA, USA), 1% Glutamax (Gibco, Carlsbad, CA, USA), 1% NEAA (Gibco, Carlsbad, CA, USA), 5 μg mL^−1^ insulin (Gibco, Carlsbad, CA, USA), and 1 μg mL^−1^ heparin (Sigma, St. Louis, MO, USA), supplemented with 5 μM SB431542 (Selleck, Houston, TX, USA, S1067) and 5 μM dorsomorphin (Selleck, Houston, TX, USA, S5900) [68]. After 8 days of induction, the cells were passaged onto new 6−well plates in N2B27 medium. Subsequently, after 16 days, canonical neural rosettes emerged and were carefully isolated as neurospheres. The neurospheres were maintained in NPC medium, supplemented with 20 ng mL^−1^ bFGF and 20 ng mL^−1^ EGF, with regular medium changes every 2 days. After 5 days of culture, these neurospheres were dissociated into single cells using Accutase and designated as P0. A total of 2.5 × 10^6^ NPCs from P0 were subjected to a proliferation assay. NPCs were passaged and counted every 6 days, resulting in the identification of P1 and P2.

EB neural specification was executed as described previously [43]. Briefly, the cells were digested with 0.5 mM EDTA. Then the cell aggregates were suspended in DMEM/F12 medium containing 20% KOSR (Gibco, Carlsbad, CA, USA), 1% Glutamax, 1% NEAA, and 0.1 mM 2-mercaptoethanol. The culture medium was refreshed daily. After 4 days, the EBs were further cultured for 3 days in N2 medium, composed of DMEM/F12 medium supplemented with 1% Glutamax, 1% NEAA, 1 × N2 and 2 μg mL^−1^ heparin. On day 7, the EBs were transferred to 10 cm plates in N2 medium plus 20% FBS, with subsequent medium changes every 2 days. After 14 days, rosette-like NPCs appeared and were resuspended in N2 medium.

Neuron spontaneous differentiation: hNPCs were dissociated into single cells and utilized for spontaneous differentiation. A total of 1 × 10^5^ hNPCs were plated onto Matrigel-coated 24-well plates and maintained in N2B27 medium containing 20 ng mL^−1^ BDNF (Peprotech, Rocky Hill, NJ, USA, 45002), 20 ng mL^−1^ GDNF (Peprotech, Rocky Hill, NJ, USA, 45010), and 1 mM cAMP (Sigma, St. Louis, MO, USA, D0260). The medium was refreshed every 2 days. After a 14-day period, typical neuronal morphology become evident, and the neurons were subjected to immunostaining analysis.

### 4.6. Quantitative Real-Time PCR (qRT−PCR)

We extracted total cellular RNA using RNAiso (TaKaRa, Kyoto, Japan) and performed reverse transcription using the PrimeScript RT reagent Kit (Perfect Real Time) (TaKaRa, Kyoto, Japan). Subsequently, qRT−PCR was conducted using SYBR qPCR Master Mix (Bio−Rad, Hercules, CA, USA) and a CFX96 machine (Bio−Rad, Hercules, CA, USA). *GAPDH* served as the internal reference for normalizing the qRT−PCR outcomes from human samples. Data analysis involved the utilization of three replicates. All primer sequences are listed in Supplementary Information Table S1 (BioSune, Shanghai, China, Supplementary Information).

### 4.7. Western Blot Analysis

Western blot was conducted following previously established procedures [69]. For the detection of METTL3 and PAX6, cell lysis was performed on ice using 1 × loading buffer (Beyotime, Shanghai, China). Whole-cell extracts were resolved by electrophoresis on SDS-PAGE gel. The resulting samples were transferred onto PVDF membranes (Millipore, Boston, MA, USA). Afterward, the PVDF membranes were blocked with 3% BSA. They were then incubated with primary antibodies at 4 °C for 12 h. Following five washes with TBST for 5 min each, the membranes were exposed to HRP-conjugated secondary antibodies. Subsequent to five additional washes with TBST for 5 min each, the membranes were subjected to detection using ECL (Beyotime, Shanghai, China) and visualization with an Amersham image analysis system (Amersham Imager 600). The antibodies were utilized in accordance with the manufacturer’s instructions for Western blot. Comprehensive antibody information is provided in Supplementary Information Table S3 (Supplementary Information).

### 4.8. Immunostaining Assay

Immunostaining was performed as described previously [24]. Cells were fixed with 4% paraformaldehyde (PFA) at room temperature for 20 min. Following three washes with PBS, cells were permeabilized with 0.3% Triton X−100 (Sigma) and blocked with 10% donkey serum in PBS, and then incubated with primary antibodies at 4 °C for 12 h. After three additional washes, the cells were treated with secondary antibodies and Hoechst 33342 (Sigma, St. Louis, MO, USA) at room temperature for 1 h. Immunostained samples were imaged using an SP-8 microscope (Leika). Comprehensive antibody information is provided in Supplementary Information Table S3 (Supplementary Information).

### 4.9. EdU Assay and Cell Cycle and Apoptosis Analyses

We conducted an EdU assay following the manufacturer’s recommendations, utilizing the Click−iT EdU Pacific Blue flow cytometry assay kit (Invitrogen, Carlsbad, CA, USA). We cultured 1 × 10^6^ METTL3^s/s^ hNPCs (with or without ASV) on 6−well plates in N2B27 medium containing 10 μM EdU for approximately 16 h. A negative control group was cultured without EdU. Subsequently, we dissociated these cells into single cells and fixed them in fixation buffer at room temperature for 20 min. Afterward, the cells were washed with PBS and permeabilized in perm/wash buffer at 4 °C for 10–15 min. After washing, we incubated the cells in PBS containing CuSO4, the fluorescent dye picolyl azide, and reaction buffer additive for 1 h at room temperature. Lastly, we analyzed the samples using a Beckman flow cytometer.

We performed cell cycle assays using a cell cycle detection kit (Keygen, Nanjing, China), according to the manufacturer’s recommendations. METTL3^s/s^ hNPCs, with and without ASV treatment, were digested to obtain single cells. Subsequently, 1 × 10^6^ cells were fixed in 70% ethanol at 4 °C overnight. After washing, the cells were incubated with binding buffer, RNase and propidium iodide (PI) at 37 °C for 30 min. Then, these cells were analyzed with Beckman flow cytometer.

We conducted apoptosis analysis utilizing an Annexin V−FITC/PI cell apoptosis detection kit (Keygen, Nanjing, China), following the manufacturer’s instructions. Briefly, 2 × 10^5^ cells were incubated with binding buffer containing Annexin V-FITC and PI at room temperature for 5–15 min. Subsequently, we analyzed the samples utilizing the Beckman flow cytometer.

### 4.10. RNA Sequencing (RNA−Seq) and Methylated RNA Imunoprecipitation Sequencing (MeRIP−Seq) and MeRIP−qPCR

Total RNAs were extracted from METTL3^s/s^ hNPCs (treated with or without ASV) using TRIzol (Invitrogen, Carlsbad, CA, USA, 15596026). DNA digestion was performed after RNA extraction using DNaseI. The quality of the RNA was evaluated by measuring the A260/A280 ratio using a Nanodrop One C Spectrophotometer (Thermo Fisher, Waltham, MA, USA). The integrity of the RNA was verified through 1.5% agarose gel electrophoresis. Quantification of the qualified RNAs were performed using Qubit3.0 with the Qubit RNA Broad Range Assay kit (Life Technologies, Carlsbad, NY, USA, Q10210).

For RNA−seq, 2 μg of total RNAs were used for the preparation of a stranded RNA-seq library using a KC Stranded mRNA Library Prep Kit for Illumina (Wuhan Seqhealth, Wuhan, China, DR08402), following the manufacturer’s instructions. PCR products with the range of 200 to 500 bps were enriched, quantified and subsequently sequenced using the Novaseq 6000 sequencer (Illumina, San Diego, CA, USA) with a PE150 model.

For MeRIP−seq, 50 μg total RNAs were utilized for polyadenylated (polyA) RNA enrichment by VAHTS mRNA Capture Beads (VAHTS, Nanjing, China, N401−01/02). 20mM ZnCl_2_ was added to mRNAs and incubated at 95 ℃ for 5min to 10min, allowing the RNA fragments of 100–200 nt. Subsequently, 10% of the RNA fragments was saved as “input”, while the remainder was employed for m^6^A immunoprecipitation (IP). The specific anti-m^6^A antibody (Synaptic Systems, German, 202203, 2.5 μg/IP) was applied for m^6^A IP. RNA samples from both the “input” and “IP” were prepared using TRIzol. The stranded RNA sequencing (MeRIP−seq) library was constructed using the KC−Digital Stranded mRNA Library Prep Kit for Illumina (Wuhan Seqhealth, Wuhan, China, DR08502), following the manufacturer’s instructions. The kit minimized duplication bias in the PCR and sequencing steps, by employing the unique molecular identifier (UMI) of 8 random bases to label the preamplified cDNA molecules. Library products in the range of 200–500 bps were enriched, quantified and ultimately sequenced on the Novaseq 6000 sequencer (Illumina, San Diego, CA, USA) with the PE150 model.

For MeRIP−qPCR, we initiated the procedure by employing total RNAs for polyA RNA enrichment using the Dynabeads mRNA Purification Kit (Thermo Fisher, Waltham, MA, USA, 61006). Following this, we introduced 20 mM ZnCl_2_ to the mRNA and incubated it at 95 °C for 5 min to 10 min, allowing the RNA fragments of 100–200 nt. Subsequently, we preserved 10% RNA fragments as “input”, allocating 40% for m^6^A IP (Abcam, Cambridge, MA, USA, ab286164, 4 μg/IP), which we designated as “IP”. The remaining 40% underwent incubation with rabbit IgG as a negative control, labeled as “IgG”. We then processed the RNA samples from “input”, “IP” and “IgG” using RNAiso. The subsequent steps followed the same procedures outlined in the qRT−PCR protocol.

### 4.11. RNA Imunoprecipitation Sequencing (RIP−Seq) and RIP−qPCR

The RIP assay was conducted on METTL3^s/s^ hNPCs by SeqHealth (Wuhan, China). The cells were treated with cell lysis buffer. A 10% portion of the sample was preserved and labeled as “input”, while 80% was used in immunoprecipitation reactions with METTL3 antibody (Abcam, Cambridge, MA, USA, ab195352, 10 μg/IP), designated as “IP”, and the remaining 10% was incubated with rabbit IgG (Cell Signaling Technology, Boston, CA, USA) as a negative control, referred to as “IgG”. RNA from the input and IP samples was extracted using TRIzol reagent (Invitrogen, Carlsbad, CA, USA, 15596026). The remaining steps were the same as those in the MeRIP-seq protocol.

For RIP−qPCR, the cells were treated with cell lysis buffer containing 100 mM KCl, 5 mM MgCl_2_, 10 mM Hepes (pH 7.0), 0.5% Nonidet P−40, 1 mM DTT, 2 μL protease inhibitor (Roche, Basel, Switzerland), and 0.5 μL RNase Out Recombinant Ribonuclease Inhibitor (Invitrogen, Carlsbad, CA, USA). The 10% lysis sample was stored as “input”, while 40% was employed in immunoprecipitation reactions with METTL3 antibody (Abcam, Cambridge, MA, USA, ab195352, 4 μg/IP) and designated as “IP”, and another 40% was subjected to incubation with rabbit IgG (Cell Signaling Technology, Boston, CA, USA) as a negative control, and referred to as “IgG”. The subsequent steps followed the same procedures outlined in the MeRIP−qPCR protocol.

### 4.12. Total RNA-Seq Data Processing and Quantification

Initially, we employed cutadapt (V1.18) to eliminate adaptor sequences, low−quality bases and reads shorter than 50 bases. Subsequently, the processed clean data were aligned to the human reference genome (hg38) using HISAT2 (V2.1.0) with the parameters described in our previous study [70]. Following this, gene expression levels were quantified as FPKM using stringtie (V1.3.4d) with the parameter “−e−rf”. Genes with FPKM < 0.1 across all samples were excluded, and FPKM values of replicates were then averaged.

### 4.13. RIP-Seq Data Processing and Analysis

Initially, RIP−seq data (METTL3 and MeRIP) were processed using the same filtering steps as RNA−seq. Afterward, the sequenced reads were subjected to base calling and demultiplexing using standard Illumina software (Illumina NovaSeq 6000), following quality control. Bowtie2 [71] was employed for aligning the reads to the hg38 reference genome. Reads mapping to the same genomic positions with identical orientation were collapsed into single reads. Peaks representing regions of m^6^A/METTL3 enrichment were identified using the Macs2 [72], with the input genomic DNA serving as a background control (parameters: W = 200; G = 600; FDR cut−off of 0.01 applied). Binding profiles and heatmaps were generated using deepTools [73]. Gene ontology analysis was conducted using the DAVID Functional Annotation Tool.

### 4.14. Statistical Analysis

Typically, the results were expressed as the mean ± SD (standard deviation), computed with Microsoft Excel and GraphPad Prism 10.0 (San Diego, CA, USA), based on a minimum of three biological repeats. Significance levels among samples were assessed using unpaired *t* tests, and one−way ANOVA or two−way ANOVA. Statistically significant differences were defined as those with a *p* value < 0.05.

## Data Availability

All data generated or analyzed during this study are included in this manuscript and its Supplementary Information files. The RNA−seq, RIP−seq and MeRIP−seq data have been deposited in the National Center for Biotechnology Information (NCBI) database under the data set identifier GSE242522.

## Supplementary Materials

The following supporting information can be downloaded at: https://www.mdpi.com/article/10.3390/ijms242115535/s1.
