# Supplementary material for: METTL3-Dependent N6-Methyladenosine Modification Programs Human Neural Progenitor Cell Proliferation
Source: Int J Mol Sci. 2023 Oct 24;24(21):15535. doi: 10.3390/ijms242115535 (PMC10647291; doi:10.3390/ijms242115535)
Supplement: Supplementary file 1 [file ijms-24-15535-s001.zip › ijms-2560612-supplementary.pdf]

Supplementary data

Inventory of Supplementary Information

Supplementary Figures and Figure Legends

Supplementary Tables

Table S1. Primers used for qRT−PCR, MeRIP−qPCR and RIP−qPCR assay.

Table S2. Primers used for vector construction.

Table S3. Antibodies used in this study.

Table S4. GO of m^6A downregulated peaks.

Table S5. GO of upregulated genes in RNA−seq.

Table S6. GO of downregulated genes in RNA−seq.

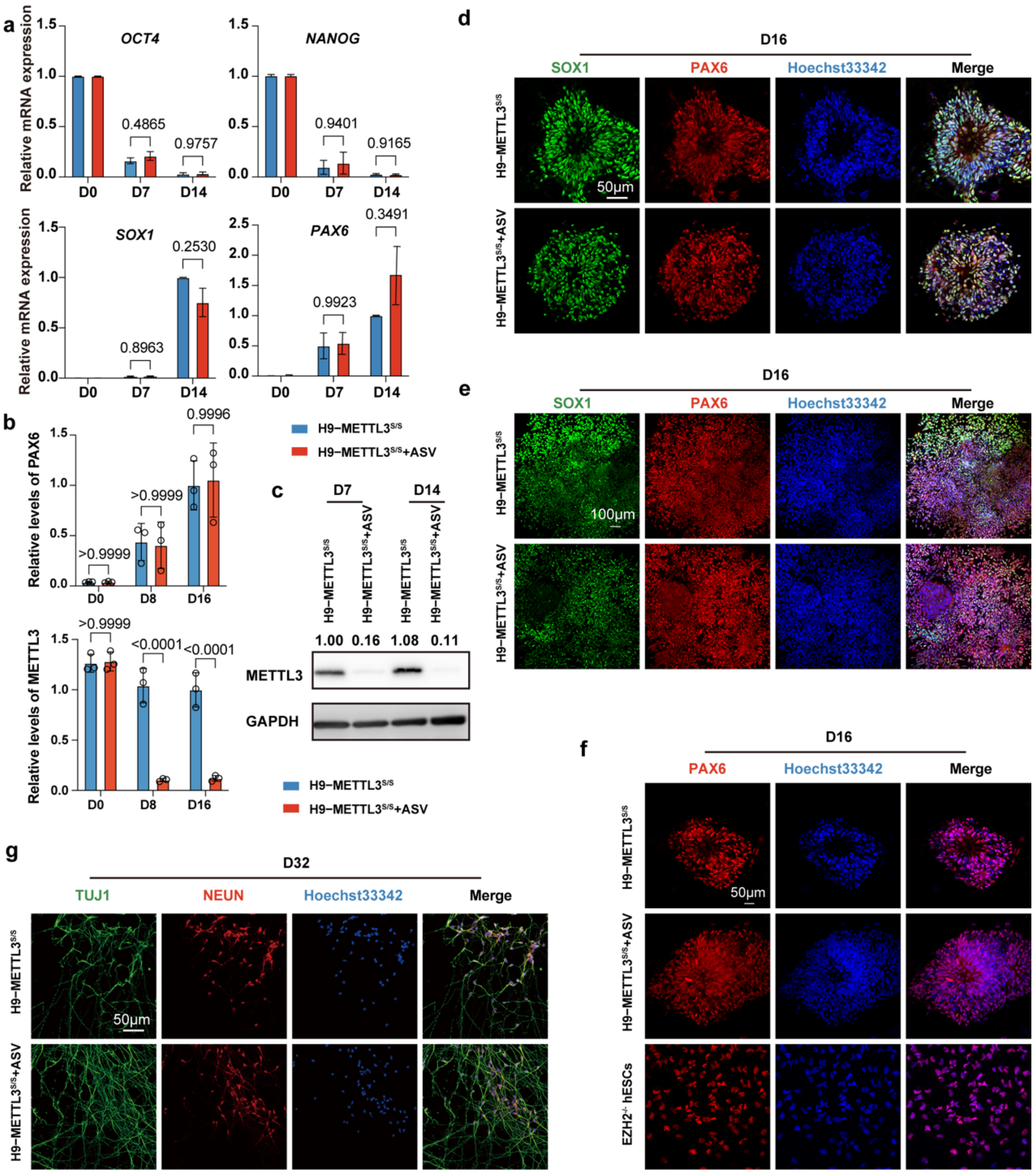

Figure S1. NPC differentiation and neuron differentiation of METTL3$^{s/s}$ hESCs using EB method. (**a**) qRT−PCR analysis on the expression level of the pluripotent genes (*NANOG* and *OCT4*) and the hNPC genes (*PAX6* and *SOX1*) in H9−METTL3$^{s/s}$ hESCs and H9−METTL3$^{s/s}$ hESCs after ASV treatment at day 0, day 7 and day 14 of neural differentiation. H9−METTL3$^{s/s}$ hESCs serve as control. Data represent mean ± SD (n = 3, two-way ANOVA). (**b**) Relative intensities analysis of western blot in Figure 2c. (**c**) Western blot of H9−METTL3$^{s/s}$ hESCs and H9−METTL3$^{s/s}$ hESCs after ASV treatment for METTL3 at day 7 and day 14 of neural differentiation. GAPDH was

probed as an internal loading control. (**d**) Immunostaining images of H9−METTL3$^{s/s}$ hESCs and H9−METTL3$^{s/s}$ hESCs after ASV treatment for SOX1 (green) and PAX6 (red) at day 16 of neural differentiation. Scale bar, 50 μm. (**e**) Immunostaining images show the rosette-like morphology of H9−METTL3$^{s/s}$ hESCs and H9−METTL3$^{s/s}$ hESCs after ASV treatment for SOX1 (green) and PAX6 (red) at day 16 of neural differentiation. Scale bar, 100 μm. (**f**) Immunostaining images show the rosette−like morphology of H9−METTL3$^{s/s}$ hESCs, H9−METTL3$^{s/s}$ hESCs after ASV treatment and EZH2$^{-/-}$ hESCs (negative control) at day 16 of neural differentiation. Scale bar, 50 μm. (**g**) Immunostaining images of H9−METTL3$^{s/s}$ hNPCs and H9−METTL3$^{s/s}$ hNPCs after ASV treatment for TUJ1 (green) and NEUN (red) at day 32 of neural differentiation. Scale bar, 50 μm.

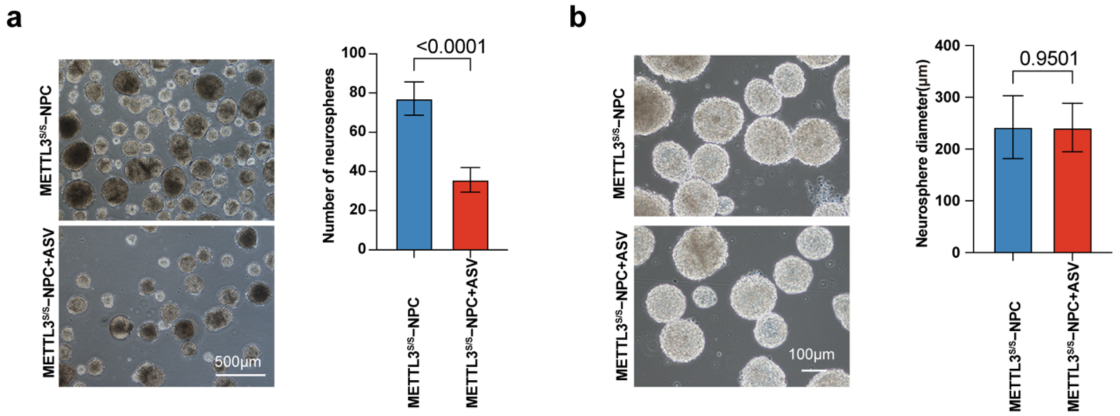

Figure S2. Characterization of METTL3$^{s/s}$ hNPCs using EB method. (**a**) The number of neurospheres in METTL3$^{s/s}$ hNPCs and METTL3$^{s/s}$ hNPCs after ASV treatment. (**b**) The diameter of neurospheres in METTL3$^{s/s}$ hNPCs and METTL3$^{s/s}$ hNPCs after ASV treatment.

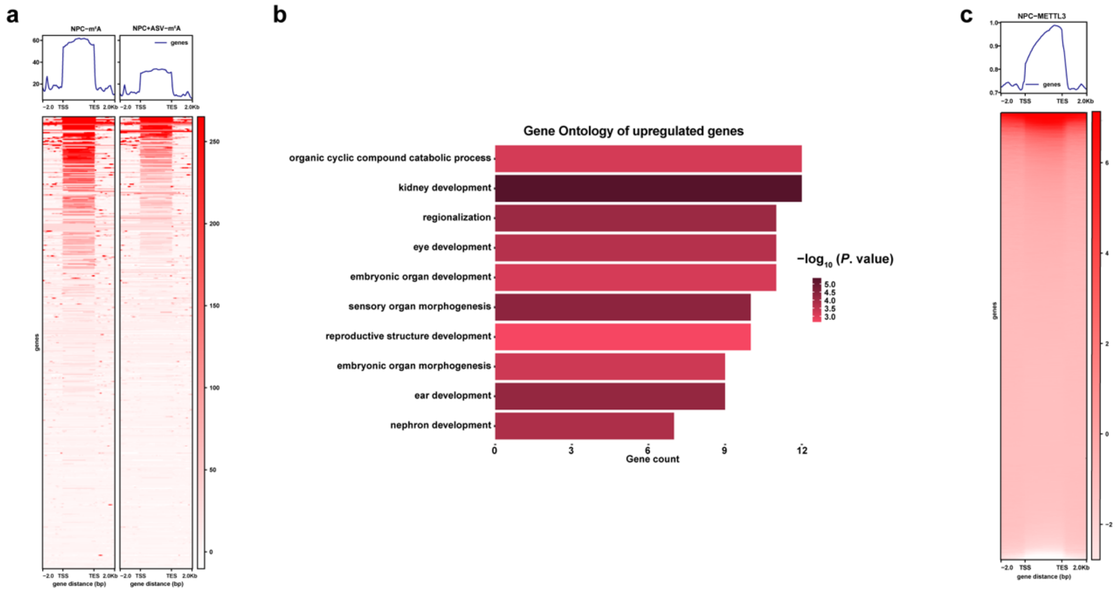

Figure S3. Bioinformatic outcomes of METTL3$^{s/s}$ hNPCs with or without ASV. (**a**) The heatmap of m^{6}A peaks in METTL3$^{s/s}$ hNPCs and METTL3$^{s/s}$ hNPCs after ASV treatment. (**b**) Gene Ontology (GO) analysis for upregulated genes in H9-METTL3$^{s/s}$ hNPCs after ASV treatment. (**c**) The heatmap of METTL3 peaks in H9-METTL3$^{s/s}$ hNPCs.

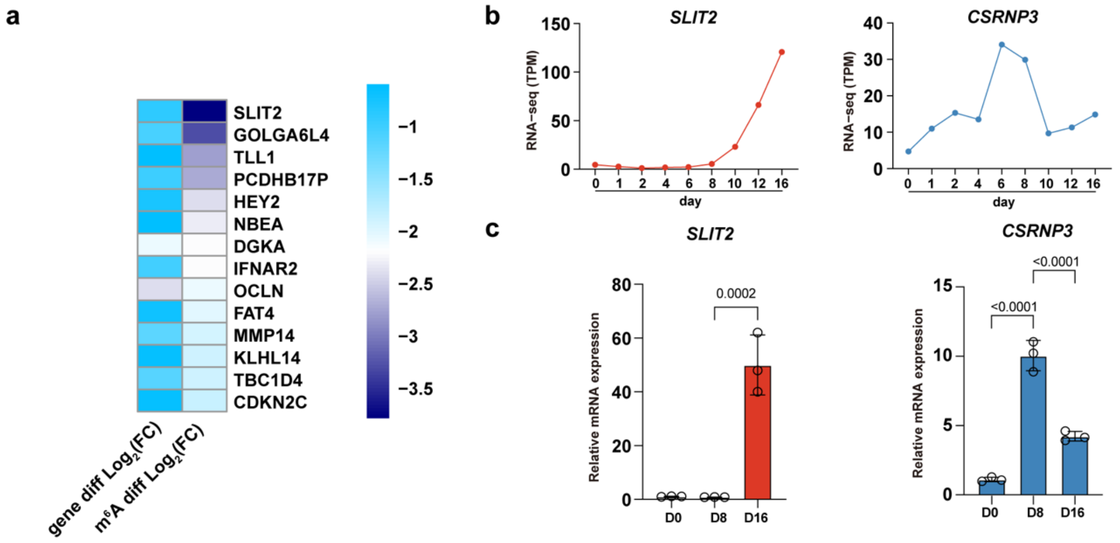

Figure S4. SLIT2 is the key gene associated with hNPCs proliferation. (**a**) The heatmap of top genes in m^{6}A differential genes and differential expression genes in METTL3$^{S/S}$ hNPCs and METTL3$^{S/S}$ hNPCs after ASV treatment. (**b**) The expression of *CSRNP3* and *SLIT2* in time-course RNA-seq. (**c**) qRT−PCR analysis on the expression level of *CSRNP3* and *SLIT2* in H9−METTL3$^{S/S}$ hESCs at day 0, day 8 and day 16 of neural differentiation. H9−METTL3$^{S/S}$ hESCs serve as control. Data represent mean ± SD (*n* = 3, two-way ANOVA).

Supplementary Tables

Table S1. Primers used for RT−qPCR, MeRIP−qPCR and RIP−qPCR assay.

hGAPDH−qF	ATGACATCAAGAAGGTGGTG	RT−qPCR
hGAPDH−qR	CATACCAGGAAATGAGCTTG	RT−qPCR
hOCT4−qF	ACATCAAAGCTCTGCAGAAA GAACT	RT−qPCR
hOCT4−qR	CTGAATACCTTCCCAAATAGA ACCC	RT−qPCR
hNANOG−qF	ATTCTTCCACCAGTCCCAAA	RT−qPCR
hNANOG−qR	ATCTGCTGGAGGCTGAGGTA	RT−qPCR
hSOX1−qF	AATTTTATTTTCGGCGTTGC	RT−qPCR
hSOX1−qR	TGGGCTCTGTCTCTTAAATTT GT	RT−qPCR
hPAX6−qF	TCTTTGCTTGGGAAATCCG	RT−qPCR
hPAX6−qR	CTGCCCGTTCAACATCCTTAG	RT−qPCR
qh−SLIT2−F2	AGCTTAGACGAATTGACCTG AGC	RT−qPCR
qh−SLIT2−R2	CCGAAGGCAGTTTATCTTGTT GG	RT−qPCR
qh−CSRNP3−F1	CTGTGACTGCCGAGTGTTCT	RT−qPCR
qh−CSRNP3−R1	ACATACGATCCACCTGGCAC	RT−qPCR
MeRIP−qPCR−SLI T2−F	TGGGAACCATTGCAACTCGG G	MeRIP−qPCR&RIP−q PCR
MeRIP−qPCR−SLI T2−R	AGCTCCTCTGTCCCTCGTGC	MeRIP−qPCR&RIP−q PCR

Table S2. Primers used for vector construction.

METTL3−sgRNA−3−Oligo1	CACCGTATGGATTCTTAGCTCTGTA
METTL3−sgRNA−3−Oligo2	AAACTACAGAGCTAAGAATCCATAC
METTL3−HL−F2	GATCTTCCGGATGGCGTGAGCAGGTTGGTGTCAAAG
METTL3−HL−R	TATGGGTATAAATTCTTAGGTTTAGAGATGATACCATCTGG
METTL3−HR−F1	ATAACTTCGTATAATGTATGCTATACGAAGTTATAAGCACTTCCTTACAGAGCTAAG
METTL3−HR−R2	ATTGTAGGAGATCTTGTGGTGGGGTTGAAGAAACTTGTTG
PURO−F1	GTACTAAATAACTTCGTATAATGTATGCTATACGAAGTTATTACCGGGTAGGGGAGGCGC
PGK−R1	ACATTATACGAAGTTATCAGGCGGGGAGGCGGCCCAAAGGGAG
SMASh−F1	GAATTTATACCCATACGATGTTCCAGATTACGCTTATCCCTACGACGTGCCTGATTATGCATACCCATATGATG
SMASh−F2	GATTATGCATACCCATATGATGTCCCCGACTATGCCGACGAGATGGAGGAGTGTTCCCAG

Table S3. Antibodies used in this study.

Antibodies	Manufacture	Catalog
METTL3 antibody	Abcam (Cambridge, MA, USA)	ab195352
SOX1 antibody	R&D system (Minneapolis, MN, USA)	AF3369
PAX6 antibody	Biolegend (San Diego, CA, USA)	901301
MAP2 antibody	Abcam (Cambridge, MA, USA)	ab32454
SOX2 antibody	R&D system (Minneapolis, MN, USA)	AF2018
NEUN antibody	Milipore (Boston, MA, USA)	ABN78
TUJ−1 antibody	Biolegend (San Diego, CA, USA)	801201
m^6A antibody	Abcam (Cambridge, MA, USA) /Synaptic Systems (German)	ab286164 /202203
Rabbit IgG	CST (Boston, CA, USA)	2729
Donkey anti−Goat IgG (H+L) Cross−Adsorbed Secondary Antibody, Alexa Fluor™ 488	Invitrogen (Carlsbad, CA, USA)	A11055
Donkey anti−Goat IgG (H+L) Cross−Adsorbed Secondary Antibody, Alexa Fluor™ 594	Invitrogen (Carlsbad, CA, USA)	A11058
Donkey anti-Rabbit IgG (H+L) Cross−Adsorbed Secondary Antibody, Alexa Fluor™ 488	Invitrogen (Carlsbad, CA, USA)	A11008
Donkey anti-Rabbit IgG (H+L) Cross−Adsorbed Secondary Antibody, Alexa Fluor™ 594	Invitrogen (Carlsbad, CA, USA)	A11012
Anti-rabbit IgG, HRP−linked Antibody	CST (Boston, CA, USA)	7074s

Table S4. GO of m^{6}A downregulated peaks.

ID	Description	pvalue	Count
GO:0008380	RNA splicing	9.11E-16	68
GO:0022613	ribonucleoprotein complex biogenesis	6.96E-12	63
GO:0016570	histone modification	2.20E-08	55
GO:0140694	non-membrane-bounded organelle assembly	1.12E-10	52
GO:0048285	organelle fission	7.63E-06	50
GO:0000280	nuclear division	1.96E-06	48
GO:0006325	chromatin organization	6.15E-07	47
GO:0044772	mitotic cell cycle phase transition	1.71E-06	47
GO:0006260	DNA replication	5.66E-13	46
GO:0007346	regulation of mitotic cell cycle	5.67E-05	45
GO:0006417	regulation of translation	0.00128771	41
GO:0050808	synapse organization	0.00038446	40
GO:0010975	regulation of neuron projection development	0.00513974	37
GO:0061564	axon development	0.01736094	36
GO:0030900	forebrain development	0.00218787	34

Table S5. GO of upregulated genes in RNA−seq.

ID	Description	pvalue	Count
GO:0001822	kidney development	4.35E-06	12
GO:1901361	organic cyclic compound catabolic process	0.00065042	12
GO:0003002	regionalization	7.49E-05	11
GO:0001654	eye development	0.00020362	11
GO:0048568	embryonic organ development	0.00066608	11
GO:0090596	sensory organ morphogenesis	4.19E-05	10
GO:0048608	reproductive structure development	0.00223968	10
GO:0043583	ear development	5.60E-05	9
GO:0048562	embryonic organ morphogenesis	0.00049445	9
GO:0072006	nephron development	0.00014847	7

Table S6. GO of downregulated genes in RNA−seq.

ID	Description	pvalue	Count
GO:0007389	pattern specification process	7.24E-10	27
GO:0061564	axon development	5.32E-07	23
GO:0034329	cell junction assembly	8.50E-07	22
GO:0030900	forebrain development	6.08E-06	19
GO:0050808	synapse organization	3.11E-05	19
GO:0016055	Wnt signaling pathway	0.00016201	18
GO:0050673	epithelial cell proliferation	0.00016753	17
GO:0030111	regulation of Wnt signaling pathway	0.00022437	15
GO:0050678	regulation of epithelial cell proliferation	0.00040783	15
GO:0030198	extracellular matrix organization	0.00078352	14
GO:0031589	cell−substrate adhesion	0.00102055	14
GO:0010631	epithelial cell migration	0.00141276	13
GO:0043410	positive regulation of MAPK cascade	0.00339217	13
GO:0098742	cell−cell adhesion via plasma−membrane adhesion molecules	0.01021831	12
GO:0050767	regulation of neurogenesis	0.03291139	12
